# Supplementary material for: Tannins amount determines whether tannase-containing bacteria are probiotic or pathogenic in IBD
Source: Life Sci Alliance. 2023 Feb 9;6(5):e202201702. doi: 10.26508/lsa.202201702 (PMC9911794; doi:10.26508/lsa.202201702)

Figure 1B

Body weight change

Days after DSS

DSS	0	1	1	1	1	1
	1	1.0211004	1.0267211	1.0081301	1.0124707	1.0058404
	2	1.0215409	1.0091703	1.004065	1.0042392	1.0180078
	3	1.0219814	1.0025887	0.995935	1.0165864	1.0078683
	4	1.0867363	0.9960072	1.0191057	1.0083549	0.9283744
	5	0.9510594	0.9595893	0.9272358	0.9215129	0.9263465
	6	0.8603145	0.9341407	0.8865854	0.8474297	0.8671317
	7	0.8796969	0.8485806	0.7882114	0.8042145	0.7174724
TA10+DSS	0	1	1	1	1	1
	1	0.9885313	0.97593	0.988644	1.0073006	1.0110097
	2	1.0142075	1.0021882	1.002004	1.0165419	1.0392837
	3	1.0142075	0.9964989	0.9663772	1.0165419	1.0144369
	4	0.9513009	0.9964989	1.000668	1.0156178	1.0264319
	5	0.9149264	0.9763676	0.9832999	0.981425	0.9968727
	6	0.8824033	0.9304158	0.9873079	0.9657148	0.977595
	7	0.8584389	0.8800875	0.8911156	1.0151557	0.852504
TA50+DSS	0	1	1	1	1	1
	1	0.9979577	0.973236	1.0049186	1.0502049	1.0199843
	2	1.021166	0.9820836	1.0261194	1.0911885	1.0522915
	3	1.0025993	0.9688122	1.0176391	1.1065574	1.0338302
	4	0.9951727	0.9480203	1.0023745	1.0901639	0.9844464
	5	0.9269402	0.89405	1.0125509	1.0881148	1.0739835
	6	0.9023394	0.9006857	1.0341757	1.0553279	0.9678313
	7	0.968251	0.8843176	0.9040027	1.1434426	0.9576776
TA250+DSS	0	1	1	1	1	1
	1	1.0123735	1.0337667	0.961786	1.0081178	1.021388
	2	1.0033746	1.0157096	0.9808708	1.0128375	0.9559144
	3	1.0096738	1.0278982	0.991079	1.0218048	0.9943256
	4	0.9898763	1.0089382	0.9839776	1.0567302	0.9620253
	5	0.9687171	0.9498761	0.9738112	0.9666194	0.842284
	6	0.9462138	0.9455487	0.8662602	0.9334486	0.8744322
	7	0.8254437	0.8891677	0.8442377	0.9047779	0.849068

Figure 1C

Colon length (cm)

	DSS day3				
Mice number	1	2	3	4	5
DSS3d	9	9.6	8.7	9.1	9.9
Pre-10TA+DSS	9.5	10	9.4	8.9	9.5
Pre-50TA+DSS	9.8	9.5	9.9	9.1	9
Pre-250TA+DSS	9.9	9.4	9.2	10	9.3
	DSS day7				
Mice number	1	2	3	4	5
DSS7d	6	6.2	5.2	5.6	6.5
Pre-10TA+DSS	6.8	6.5	7.2	6.9	7.4
Pre-50TA+DSS	7.4	7.5	8.6	8.3	7
Pre-250TA+DSS	7.1	6.5	7.2	6.7	6.9

Figure 1D

Histological score

	Mouse NO.	1	2	3	4	5
Day 3	DSS	0	0	1	0	0
	TA10+DSS	0	1	1	0	0
	TA50+DSS	0	0	0	1	0
	TA250+DSS	2	1	3	3	2
Day 7	DSS	6	5	6	6	5
	TA10+DSS	5	5	4	3	4
	TA50+DSS	1	2	2	1	3
	TA250+DSS	6	6	6	6	5

Figure 1E

		Fold of change relative to GAPDH				
IL-1beta	DSS	2.099433	2.099433	0.846745	0.790041	0.707107
	TA50+DSS	0.779165	0.205898	0.239816	0.366021	0.48971
IL-6	DSS	1.853176	1.931873	1.180993	0.926588	0.482968
	TA50+DSS	0.146604	0.04095	0.036147	0.15283	0.185565
TNF-a	DSS	1.134455	1.241427	0.802181	1.001387	1.001387
	TA50+DSS	0.743291	0.75891	0.883928	0.934327	0.791137
Cxcl1	DSS	1.837826	1.19582	0.833931	0.725979	0.751581
	TA50+DSS	0.573554	0.602069	0.327145	0.36299	0.370617
Cxcl2	DSS	1.306765	1.004168	1.039579	0.936921	0.782412
	TA50+DSS	0.462011	0.462011	0.391206	0.357496	0.247586

Figure 1F GAPDH

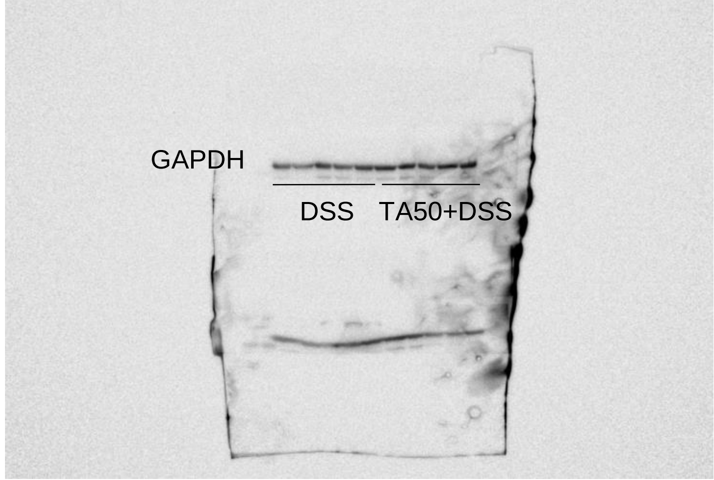

Figure 1F p-stat3, p-ERK and p-p65

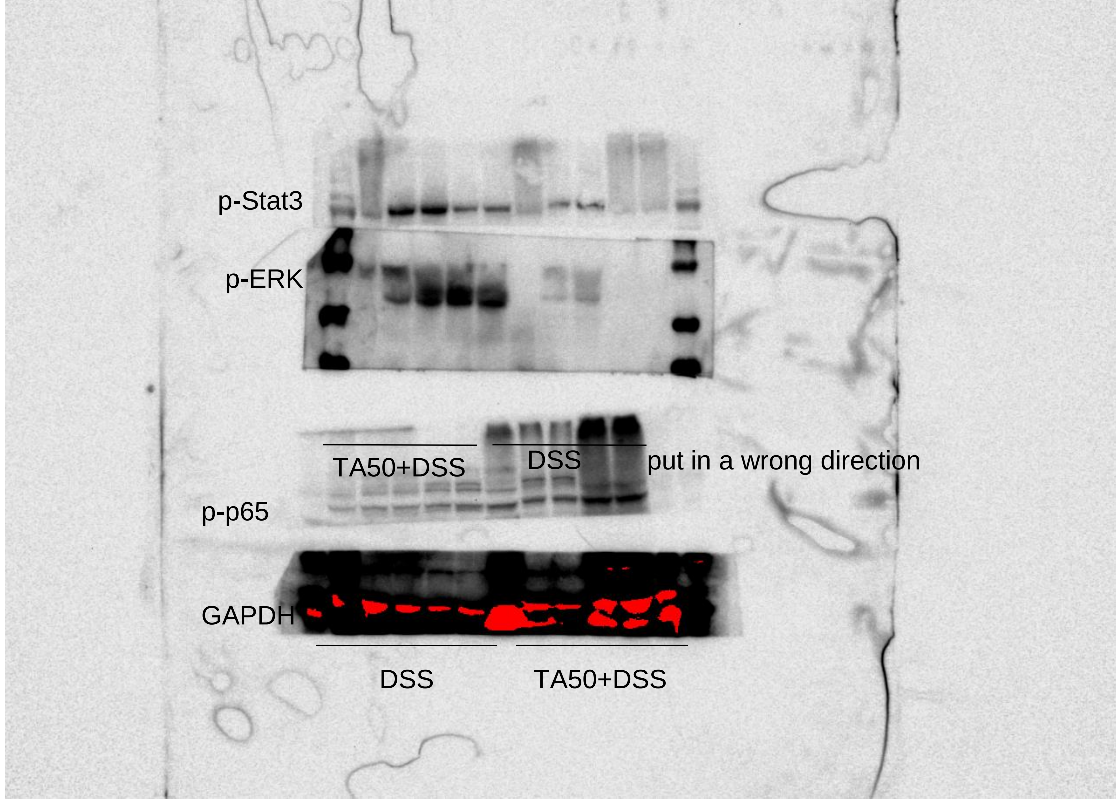

Supplement: Supplementary file 1 [file LSA-2022-01702_SdataF1.pdf]
