# Supplementary material for: Tannins amount determines whether tannase-containing bacteria are probiotic or pathogenic in IBD
Source: Life Sci Alliance. 2023 Feb 9;6(5):e202201702. doi: 10.26508/lsa.202201702 (PMC9911794; doi:10.26508/lsa.202201702)
Supplement: Supplementary file 2 [file LSA-2022-01702_SdataF2.pdf]

Figure 2C

OD490

Day0							
Control		0.1752	0.1753	0.1603	0.1439	0.1234	0.1173
GA (ug/ml)	0	0.185	0.174	0.1627	0.1529	0.1354	0.1211
	100	0.21	0.1892	0.1701	0.1692	0.1513	0.1223
	50	0.1923	0.1743	0.1636	0.1578	0.1368	0.121
	20	0.2125	0.194	0.1772	0.1578	0.1468	0.1272
	10	0.2119	0.1988	0.1816	0.1624	0.1472	0.1292
	5	0.197	0.1872	0.1829	0.1556	0.1459	0.1361
	2.5	0.1961	0.1793	0.1634	0.1477	0.1375	0.1297
	1.2	0.1852	0.1811	0.1691	0.1371	0.1203	0.1254
	0.6	0.2002	0.2011	0.1817	0.1558	0.14	0.1279
	0.3	0.2382	0.1949	0.1754	0.1657	0.1625	0.1568
	0.15	0.2171	0.2146	0.1703	0.1666	0.1542	0.1457

Day1							
Control		0.3293	0.3075	0.2518	0.2539	0.2503	0.2742
GA (ug/ml)	0	0.3386	0.2791	0.2366	0.2366	0.2313	0.2574
	100	0.1227	0.1199	0.1167	0.1125	0.1095	0.105
	50	0.1779	0.1836	0.1343	0.1149	0.1508	0.1446
	20	0.2859	0.2487	0.2189	0.2004	0.2292	0.2237
	10	0.3043	0.2523	0.2319	0.2041	0.2266	0.2503
	5	0.3299	0.2867	0.2594	0.2631	0.2446	0.2574
	2.5	0.3227	0.2966	0.2809	0.2873	0.2739	0.2543
	1.2	0.3602	0.2239	0.2675	0.2374	0.2541	0.2378
	0.6	0.2994	0.2065	0.264	0.2655	0.2572	0.296
	0.3	0.3071	0.2147	0.2653	0.2764	0.2698	0.2434
	0.15	0.3404	0.2385	0.2579	0.2705	0.2968	0.3215

Day2							
Control		0.5184	0.449	0.3453	0.3545	0.4066	0.4529
GA (ug/ml)	0	0.2777	0.293	0.2818	0.3016	0.3161	0.3184
	100	0.1368	0.1342	0.1291	0.1374	0.1235	0.1308
	50	0.2274	0.2145	0.2241	0.2075	0.2564	0.2541
	20	0.3388	0.326	0.3437	0.3406	0.3575	0.3959
	10	0.3441	0.3309	0.3571	0.3609	0.3363	0.3963
	5	0.3234	0.3273	0.2944	0.2852	0.334	0.3497
	2.5	0.3356	0.3298	0.3023	0.3001	0.2172	
	1.2	0.2998	0.304	0.2702	0.2875	0.3327	0.2545
	0.6		0.3355	0.3099	0.2808	0.3248	0.2914
	0.3		0.2828	0.3285	0.2918	0.3088	0.2972
	0.15		0.2832	0.3255	0.3202	0.3536	0.2684

Figure 2E

Tannase activity (U/g)						
Comtral	30.6	0	0	0	0	59
TA50	84.1	214	109	965	1259	
TA250	1392	1399	1848	1644	1511	1133

Figure 2F

Colon tissue (ug/0.1g tissue)			
	Mouse 1	Mouse 2	Mouse 3
GA0	0	0	0.1795672
GA50	0.6803096	0.3902161	0.3526921
GA250	1.081272	1.6517032	2.4556365

Feces (ug/0.1g feces)			
	Mouse 1	Mouse 2	Mouse 3
GA0	0.0025463	0.0074338	0.0014276
GA50	0.3652352	0.2482473	0.2622723
GA250	0.8401149	0.7068388	0.5031606
