# Supplementary material for: Tannins amount determines whether tannase-containing bacteria are probiotic or pathogenic in IBD
Source: Life Sci Alliance. 2023 Feb 9;6(5):e202201702. doi: 10.26508/lsa.202201702 (PMC9911794; doi:10.26508/lsa.202201702)

Figure 3B

Body weight change

Days after DSS

DSS

Days after DSS					
0	1	1	1	1	1
1	0.9357798	1.12844	1.19171	0.9570816	1.030769
2	1.089109	0.8947368	1.057692	0.962963	1
3	0.963366	1.02037	0.9315069	1.022222	0.9708334
4	0.957346	0.939896	0.972165	0.904854	0.949724
5	0.9018584	0.935135	0.9647577	0.95	0.909005
6	0.87175	0.9136364	0.8025424	0.8404255	0.860606
7	0.849495	0.8023923	0.815075	0.801511	0.817033

GA10+DSS

0	1	1	1	1	1
1	0.930131	1.1	0.9856459	0.932	1.111111
2	1.173267	1.089109	0.9761062	1.027149	0.9208333
3	0.950485	0.9614718	0.997541	0.9711934	0.9416667
4	0.9701493	0.939409	0.9481132	0.9454545	1.018957
5	0.9375708	0.9717352	0.904292	0.9255102	0.9466135
6	0.869204	0.8107143	0.8529412	0.902449	0.882266
7	0.830928	0.8065854	0.852941	0.8855072	0.921175

GA50+DSS

0	1	1	1	1	1
1	1.122066	1.007905	1.150485	0.9184549	0.926087
2	0.9662447	1.090909	1.025253	0.9942731	1.03198
3	0.959072	0.960302	1.003927	0.9959322	0.9666372
4	0.960256	0.969479	0.989204	0.9501923	0.920934
5	0.9558883	0.9506977	0.9418803	0.9861789	0.909787
6	0.9427039	0.946275	0.948374	0.920354	0.914276
7	0.931679	0.927436	0.901574	0.885892	0.831691

GA250+DSS

0	1	1	1	1	1
1	1.037657	0.9992157	0.9818566	1	1.084507
2	0.9975983	0.9291667	0.952709	1	1.026906
3	0.9326693	0.923223	0.9828326	0.976	0.9843568
4	0.971066	0.9342723	0.9055794	0.924	0.9596681
5	0.9290323	0.933613	0.919797	0.968807	0.9140496
6	0.876923	0.8545454	0.9011111	0.914423	0.8875
7	0.821564	0.8042268	0.8264706	0.8911917	0.891362

Figure 3C

Colon length (cm)

DSS day3					
Mouse NO.	1	2	3	4	5
GA0	7.7	7.6	6.9	7.6	7.4
GA10	7.5	7.5	7.1	7.8	8.2
GA50	8	8.2	7.9	8.5	8.6
GA250	7.2	8.4	8.2	7.7	8.3

DSS day7					
Mouse NO.	1	2	3	4	5
GA0	5.9	5.7	5.5	4.9	4.5
GA10	4.5	6	4.3	5.3	5.5
GA50	6.1	6.1	6.3	5.8	5.7
GA250	4	4.2	5	5.1	4.2

Figure 3D

Histological score

DSS day3					
Mouse NO.	1	2	3	4	5
GA0	0	1	0	0	0
GA10	0	0	1	0	0
GA50	0	0	1	0	0
GA250	3	2	2	3	2

DSS day7					
Mouse NO.	1	2	3	4	5
GA0	6	6	6	5	6
GA10	6	5	4	4	4
GA50	3	2	2	2	1
GA250	6	6	6	5	6

Figure 3E

		Fold of change relative to GAPDH				
IL-6	DSS	0.959264	0.835088	1.06437	0.965936	1.117287
	GA50+DSS	0.48971	1.035265	0.535887	0.550953	0.697372
CCx12	DSS	1.145518	0.516199	0.572759	2.243887	1.315855
	GA50+DSS	0.122089	0.081109	0.141218	0.092526	0.082813
CCx11	DSS	1.129747	0.761017	1.271032	0.761017	0.976709
	GA50+DSS	0.719966	0.690637	0.898755	0.635516	0.322194
IL-1β	DSS	1.486583	1.207481	1.549712	0.441964	0.813379
	GA50+DSS	1.008352	0.2469	0.188417	0.463937	0.295658
TNF-α	DSS	1.125058	0.729005	1.251796	0.729005	1.167967
	GA50+DSS	0.210808	0.860949	0.508387	0.89131	0.448755

Figure 3F p-p65

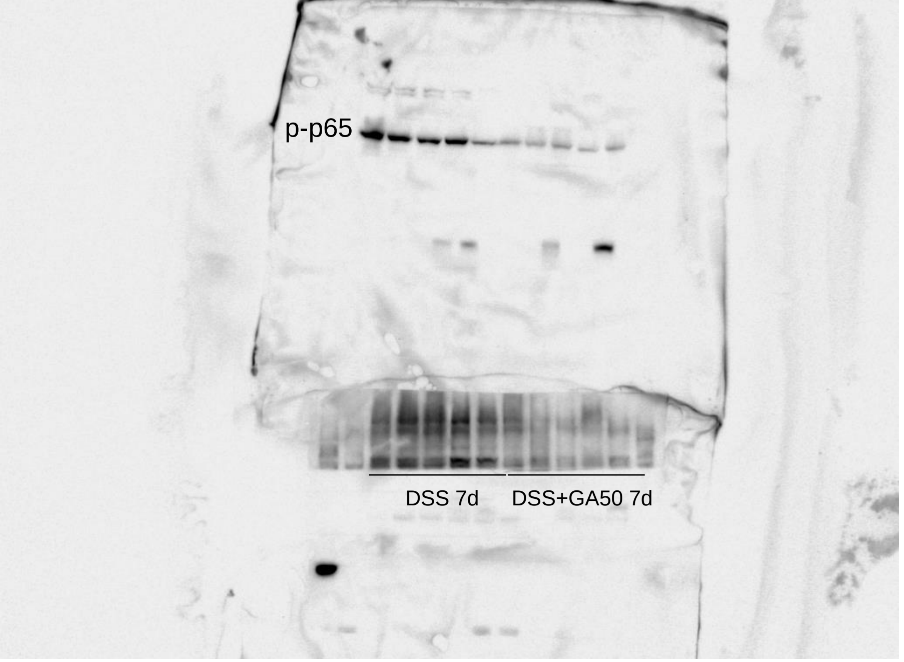

Figure 3F p-ERK

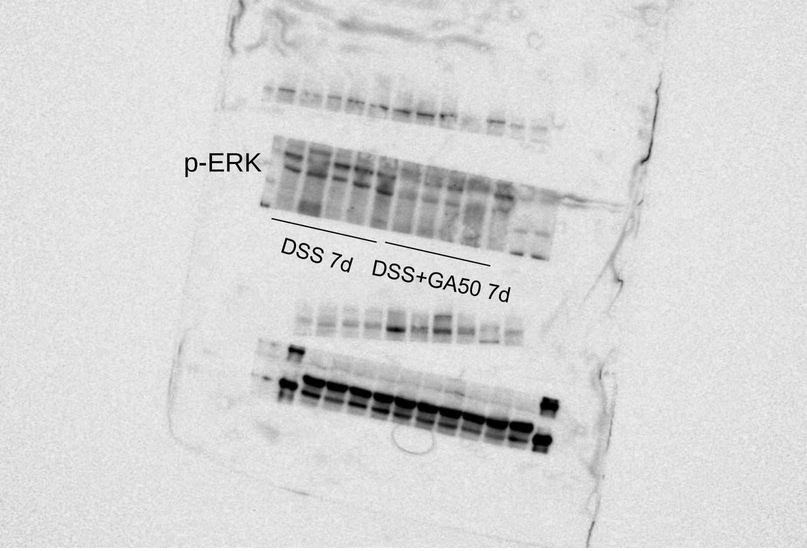

Figure 3F GAPDH

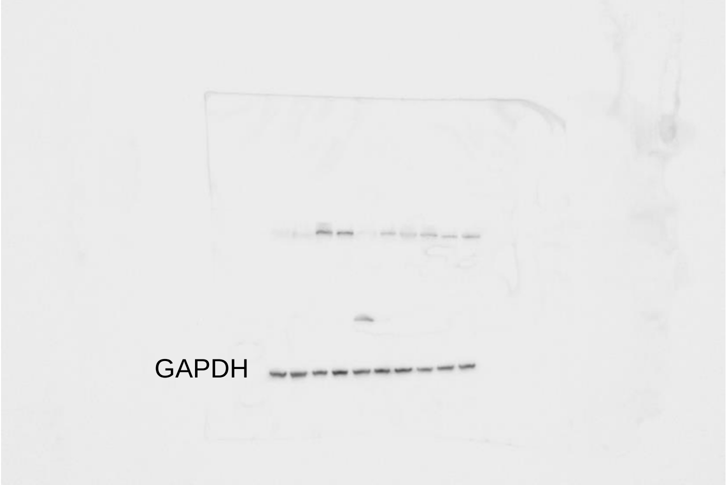

Figure 3F p-stat3

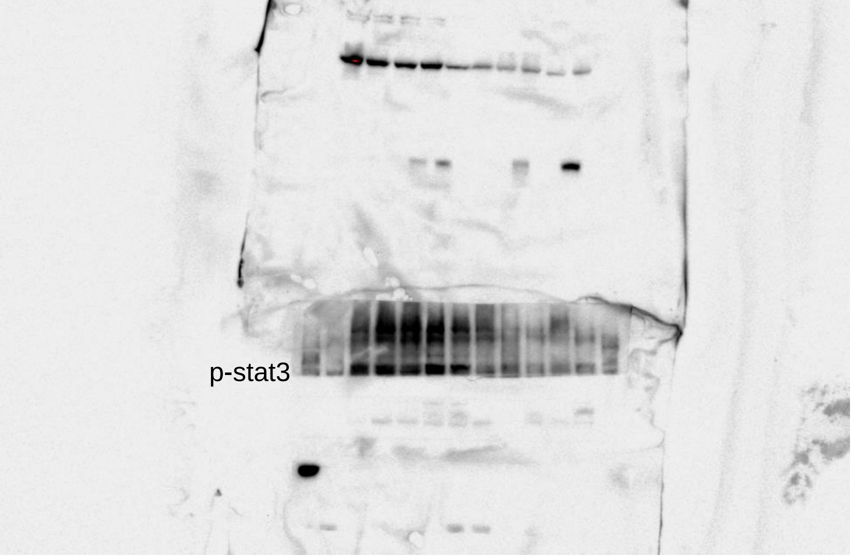

Figure 3G
GAPDH

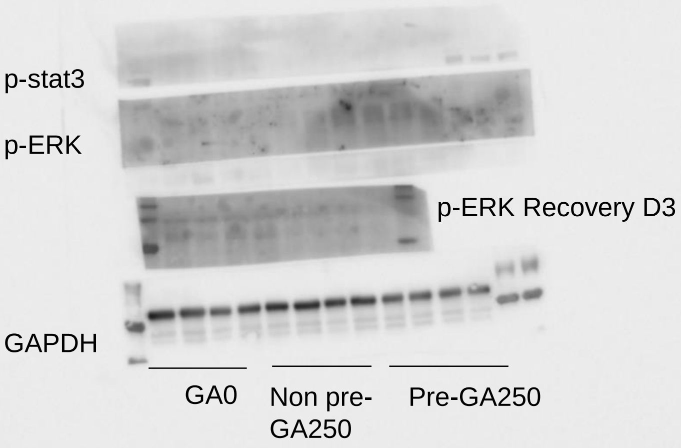

Figure 3G
pERK

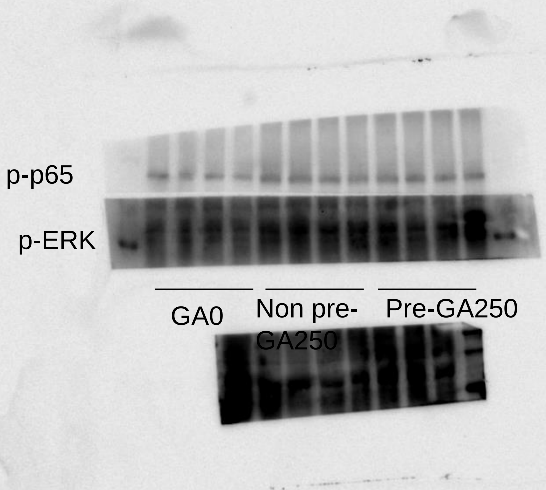

Figure 3G
p-p65

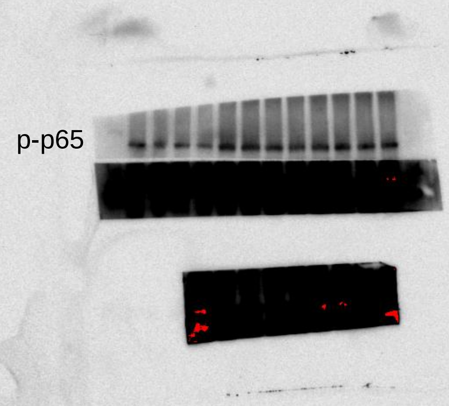

Figure 3G
p-stat3

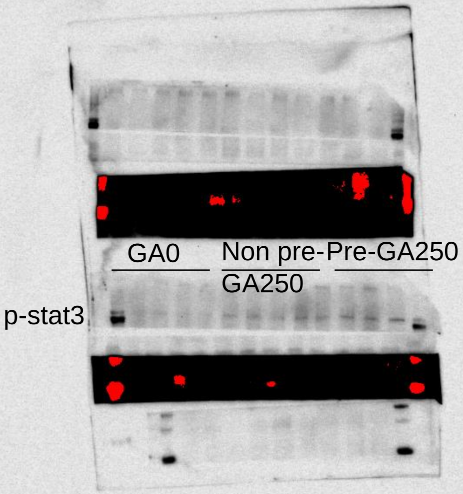

Supplement: Supplementary file 4 [file LSA-2022-01702_SdataF3.pdf]
