# Supplementary material for: Tannins amount determines whether tannase-containing bacteria are probiotic or pathogenic in IBD
Source: Life Sci Alliance. 2023 Feb 9;6(5):e202201702. doi: 10.26508/lsa.202201702 (PMC9911794; doi:10.26508/lsa.202201702)
Supplement: Supplementary file 6 [file LSA-2022-01702_SdataF4.pdf]

Figure 4 B

		Mouse 1	Mouse 2	Mouse 3	Mouse 4	Mouse 5
Death time	GA0	5	5	5	5	5
Day after DSS	GA50	7	7	6	6	6
treatment	GA250	4	5	5	5	5

Histological score

Figure 4 C

		Mouse 1	Mouse 2	Mouse 3	Mouse 4	Mouse 5
Day5	DSS	3	3	4	3	3
	GA50+DSS	2	2	2	1	3
Day3	DSS	1	2	1	1	1
	GA250+DSS	2	2	3	2	2
