# Supplementary material for: Tannins amount determines whether tannase-containing bacteria are probiotic or pathogenic in IBD
Source: Life Sci Alliance. 2023 Feb 9;6(5):e202201702. doi: 10.26508/lsa.202201702 (PMC9911794; doi:10.26508/lsa.202201702)

Figure 5B

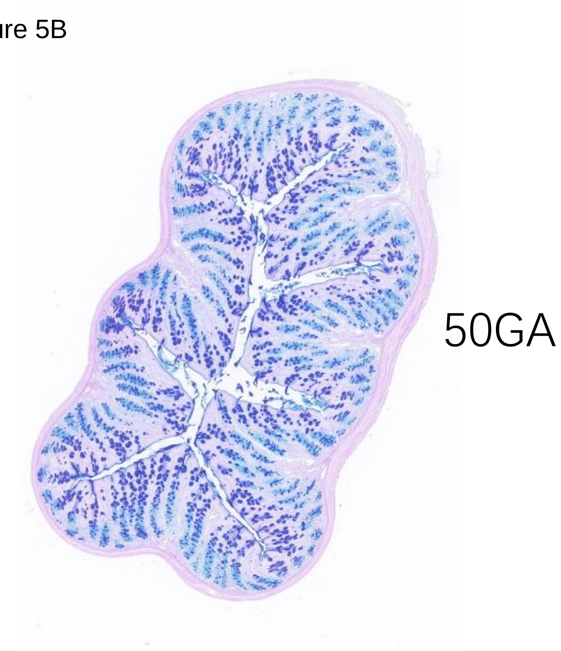

50GA

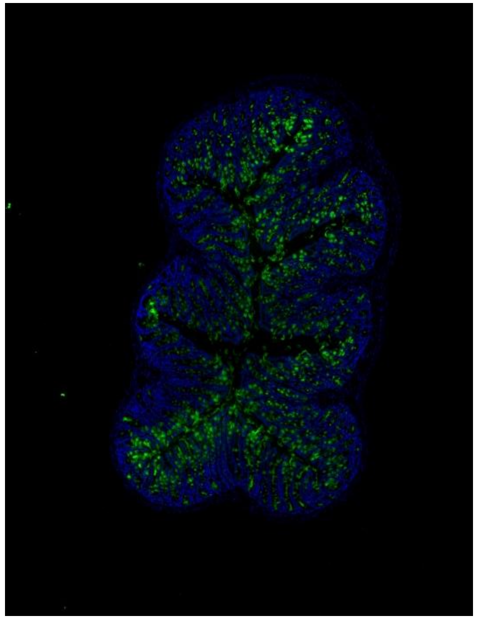

Goblet cells/cript

	Mouse 1				
GA50	21	17	15	8	9
GA0	11	5	7	14	6
	Mouse 2				
GA50	23	18	8	14	16
GA0	8	3	5	8	9
	Mouse 3				
GA50	15	18	11	9	7
GA0	4	5	2	6	3

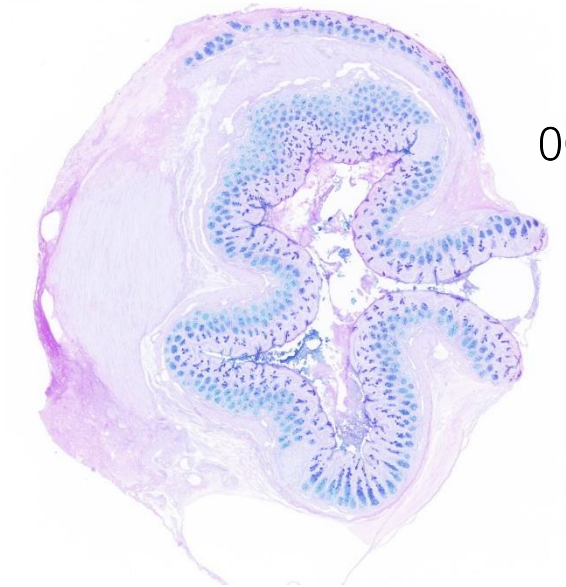

0GA

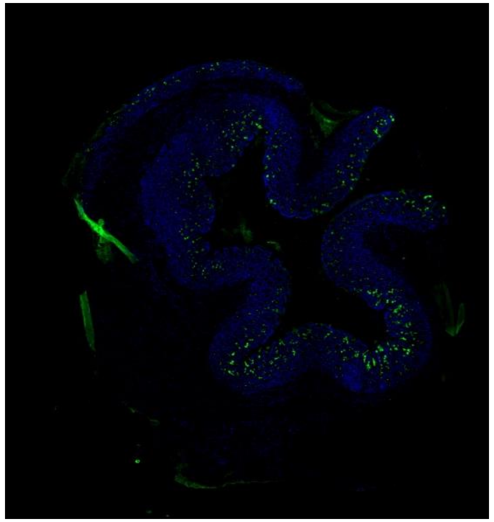

Figure 5D

Goblet cells/cript

		Mouse 1				
DSS 3 Day	GA250+DSS	3	8	14	4	5
	DSS	0	0	2	4	5
		Mouse 2				
	GA250+DSS	8	9	11	13	14
	DSS	7	3	4	2	5
		Mouse 3				
	GA250+DSS	9	9	6	7	9
	DSS	3	3	2	0	4

Figure 5C

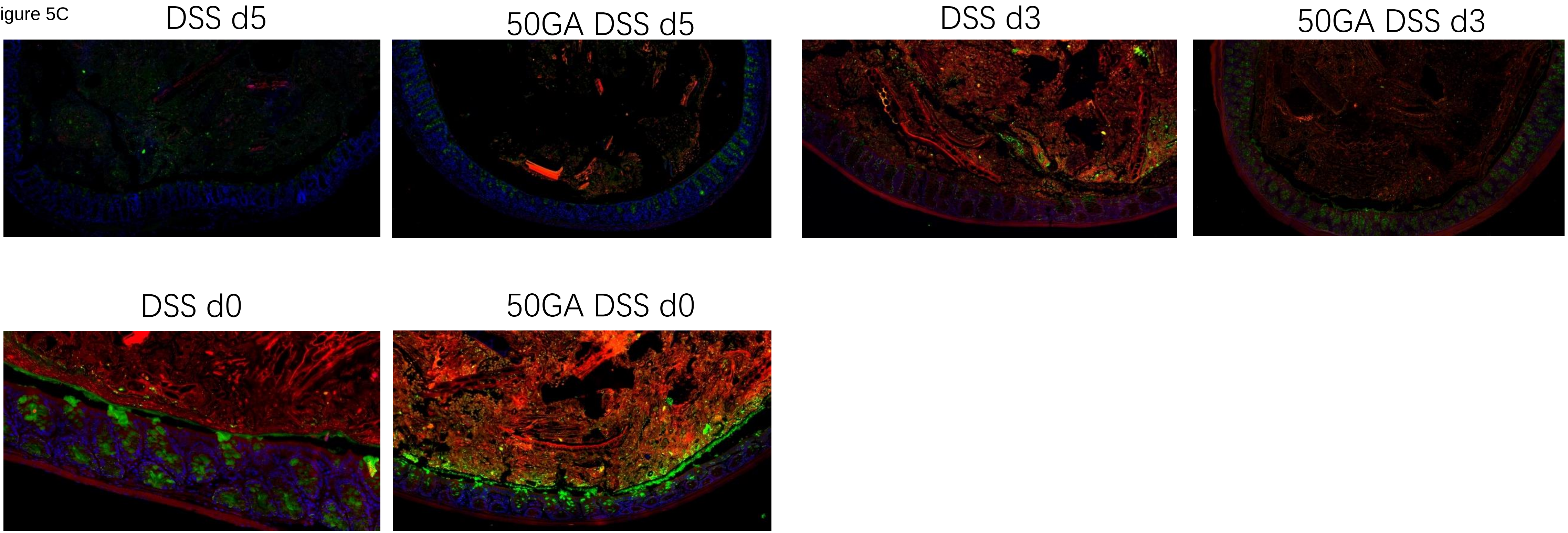

Thickness (um)

	GA50														
	Mouse 1					Mouse 2					Mouse 3				
Day0	120	100	80	110	123	118	85	74	69	121	114	93	64	59	103
Day3	21	35	17	25	29	14	22	40	35	42	16	21	43	22	34
Day5	0	0	0	0	0	0	0	0	0	0	0	0	0	0	0

	GA0														
	Mouse 1					Mouse 2					Mouse 3				
Day0	54	42	37	74	55	42	81	32	48	45	29	68	75	25	43
Day3	0	0	6	2	0	6	2	0	0	0	0	0	0	0	0
Day5	0	0	0	0	0	0	0	0	0	0	0	0	0	0	0

Figure 5F

		Fold of change relative to GAPDH					
IL-6	GA0	0.520511	0.783497	1.264003	1.100379	1.76296	
	GA250	0.682074	0.93174	1.108032	1.048262	0.706127	0.893785
CCx12	GA0	2.170459	0.602069	1.070289	0.456283	1.566994	
	GA250	10.76294	7.351303	5.456591	4.164086	12.97803	9.050516
CCx11	GA0	1.670176	1.60214	0.63728	0.716978	0.817902	
	GA250	5.241574	1.647182	1.453973	2.099433	5.133704	3.24901
IL-1β	GA0	1.41814	1.745935	0.49107	0.903753	0.910039	
	GA250	1.9917	1.562656	3.103723	2.895876	4.419858	4.544117
TNF-α	GA0	0.652477	0.885154	1.143931	0.797745	1.897369	
	GA250	3.516158	3.326488	0.910039	1.770308	4.512729	4.870264

Figure 5G

GA250 30d

GA 0 30d

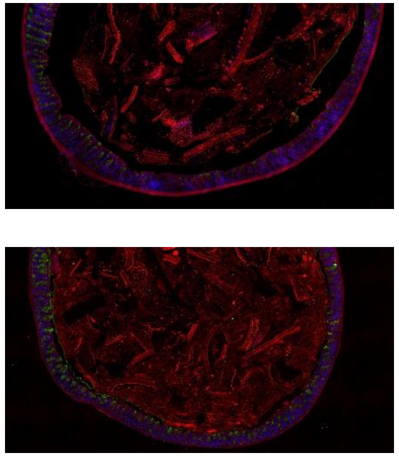

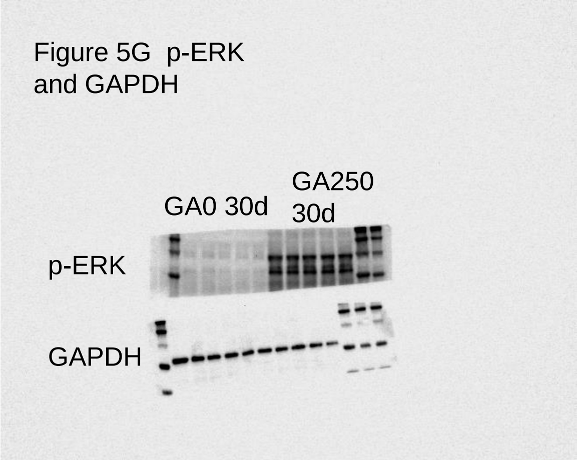

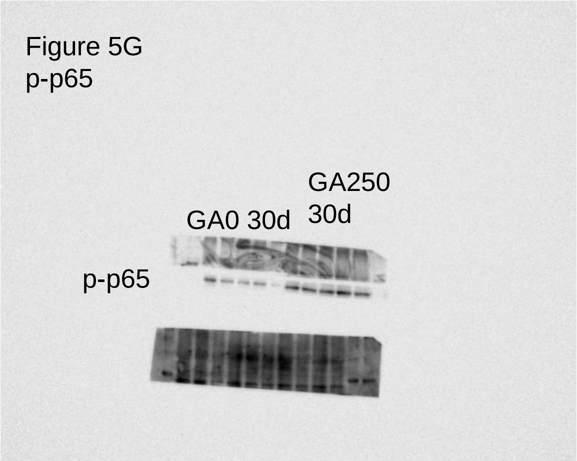

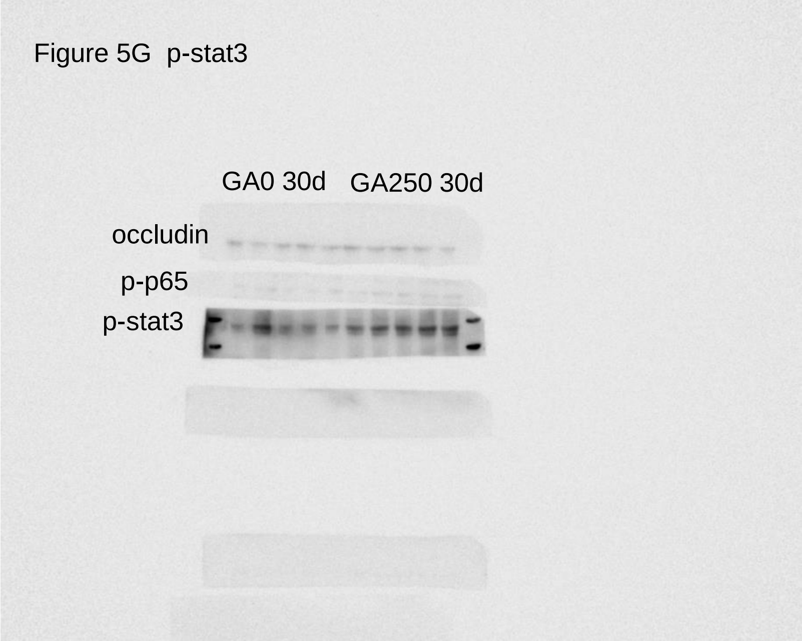

Supplement: Supplementary file 7 [file LSA-2022-01702_SdataF5.pdf]
