# Supplementary material for: Tannins amount determines whether tannase-containing bacteria are probiotic or pathogenic in IBD
Source: Life Sci Alliance. 2023 Feb 9;6(5):e202201702. doi: 10.26508/lsa.202201702 (PMC9911794; doi:10.26508/lsa.202201702)
Supplement: Supplementary file 8 [file LSA-2022-01702_SdataF6.pdf]

Figure 6D

OD490

Control	0.2242	0.2112	0.3119	0.2244	0.2281	0.3078	0.2456	0.2546
DSS	0.1358	0.1195	0.1032	0.153	0.1919	0.1292	0.1583	0.1974
DSS+PFTα 1μM	0.2281	0.1906	0.165	0.1664	0.1741	0.2288	0.1413	0.2163
DSS+PFTα 0.5μM	0.2423	0.1317	0.2142	0.1881	0.2186	0.2113	0.2026	0.2365

Figure 6F

		Sample 1	Sample 2	Sample 3
G0/G1	Control	69.7	73.1	69.8
	GA50	55.3	54.4	64.5
S	Control	5.34	4.88	5.21
	GA50	15.7	16.5	13.4
G2/M	Control	17.8	16.3	13.7
	GA50	21.4	20.6	15.4

Figure 6C
p-p53

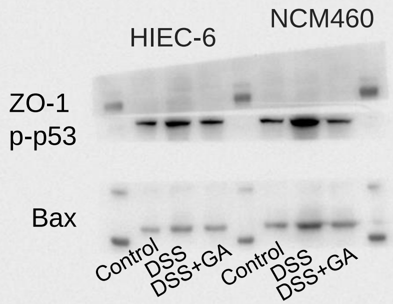

Figure 6C
Bax

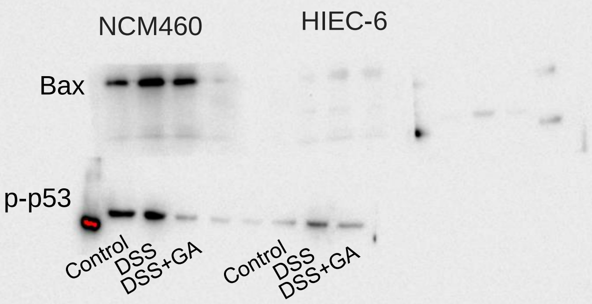

Figure 6C
BCl2

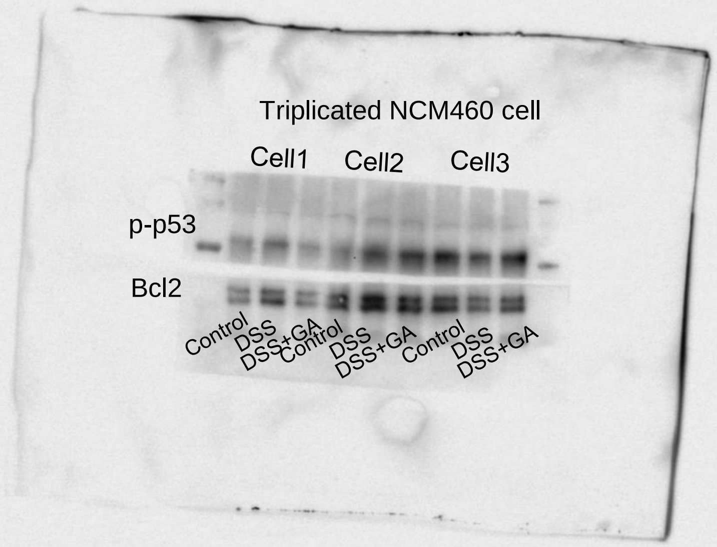

Figure 6C
p53

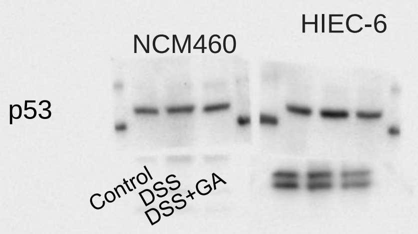

Figure 6C and Figure 7E GAPDH

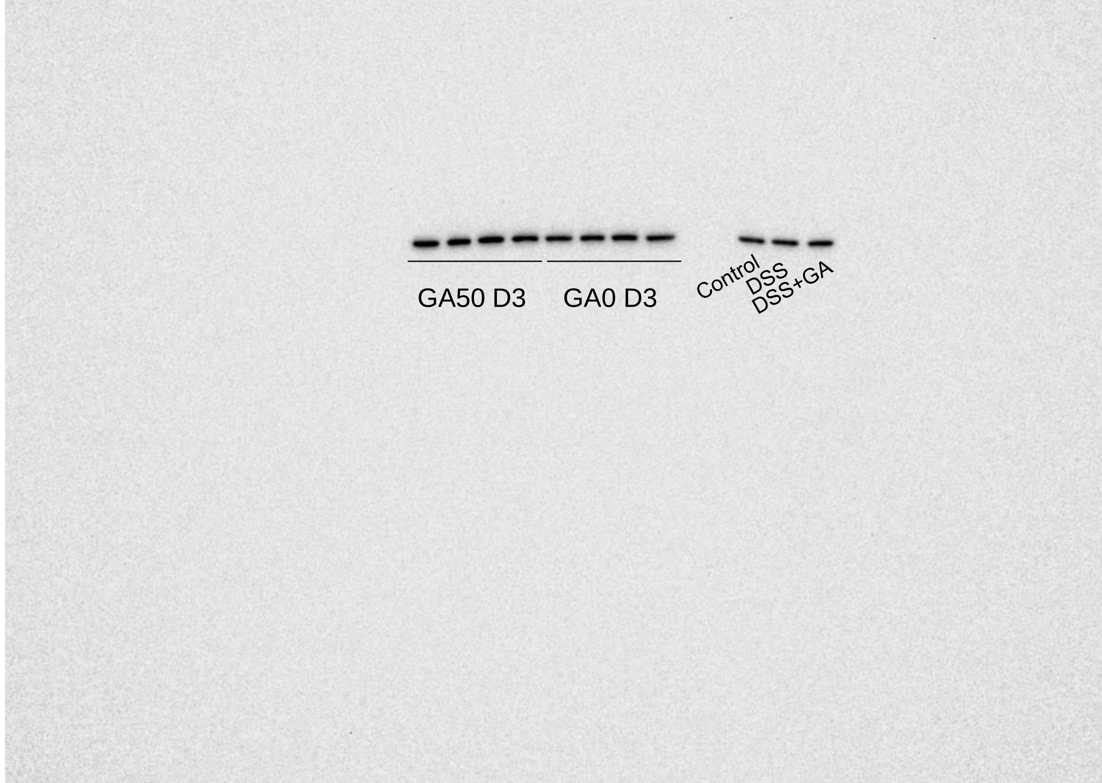

Figure 6G

Fold of change relative to beta-actin

	Control			GA50ug/ml		
**CCNB1**	0.955641	1.03178	1.012582	0.387202	0.389154	0.331888
PLK1	0.973079	1.032173	0.994747	0.35916	0.366382	0.365069
**CDC20**	0.948601	1.052397	0.999001	0.425808	0.475118	0.487105
**CCNB2**	0.965016	1.031216	1.003767	0.456943	0.444564	0.435414
CCNA2	0.941964	1.03373	1.024306	0.483631	0.510913	0.414187
**BUB1B**	0.979224	0.951524	1.069252	0.480609	0.514543	0.468144
PRKDC	1.075569	0.957667	0.966765	0.532391	0.515414	0.436437
PTTG1	0.914983	1.059092	1.025924	0.509531	0.517537	0.528974
BUB1	1.012917	1.014254	0.972828	0.537194	0.485746	0.38686
CDC25C	1.004337	0.957502	1.038161	0.522984	0.426713	0.411102
TGFB2	1.181818	0.909091	0.981522	0.381818	0.452213	0.347787
