# Supplementary material for: Tannins amount determines whether tannase-containing bacteria are probiotic or pathogenic in IBD
Source: Life Sci Alliance. 2023 Feb 9;6(5):e202201702. doi: 10.26508/lsa.202201702 (PMC9911794; doi:10.26508/lsa.202201702)

Figure 7B

Body weight change

GA50															
Day1	0.955	0.94	0.911	0.95	0.899	0.962	0.972	1.03	0.929	0.939	0.987	0.975	1.018	0.861	0.871
Day2	0.909	0.88	0.839	0.879	0.812	0.918	0.937	1.018	0.888	0.901	0.94	0.925	0.994		
Day3	0.871	0.833	0.815	0.83		0.899	0.895	1.036	0.87	0.873	0.96	0.944	1.031		
Day4	Kill	Kill	Kill	Kill		0.975	0.951	1.073	0.935	0.854	0.902	0.948	1.036		
Day5						1.051	1	1.073	0.982	0.891	0.985	1.033	1.091		
Day6						1.07	1.021	1.085	0.97	1.022	1.014	1.019	1.064		
Control															
Day1	0.887	0.974	0.888	0.914	0.914	0.866		0.855	0.972	0.91	0.855	0.852	0.858	0.994	0.892
Day2	0.768	0.91	0.783	0.816	0.871			0.809	0.852	0.813				1	
Day3	0.735	0.855		0.85				0.796	0.817	0.771				0.959	
Day4	0.834	0.975		0.986				Kill	Kill	Kill				Kill	
Day5	0.899	1.037		1.122											
Day6	0.935	1.087		1.167											

Figure 7C

Histological score

Day0	Control	6	6	6	6
	GA0	5	6	5	6
Day3	GA50	2	3	2	3

Figure 7D

Fold of change relative to GAPDH

		Mosue1	Mosue2	Mosue3	Mosue4
IL-1β	GA50	1.109569	1.414214	0.90125	0.807107
	GA0	0.795536	0.602904	0.650671	0.80107
IL-6	GA50	1.12701	1.650039	0.987943	0.54431
	GA0	0.200615	0.08612	0.15416	0.216509
TNFα	GA50	0.9726549	0.882703	1.1095695	1.057018
	GA0	0.6155722	0.652638	0.7022224	0.7659363
Cxcl1	GA50	1.368409	1.882304	0.84431	0.71326
	GA0	0.483806	0.379586	0.281752	0.37436
Cxcl2	GA50	0.942785	1.21841	0.709562	1.226885
	GA0	0.078292	0.056917	0.184923	0.149167

Figure 7E

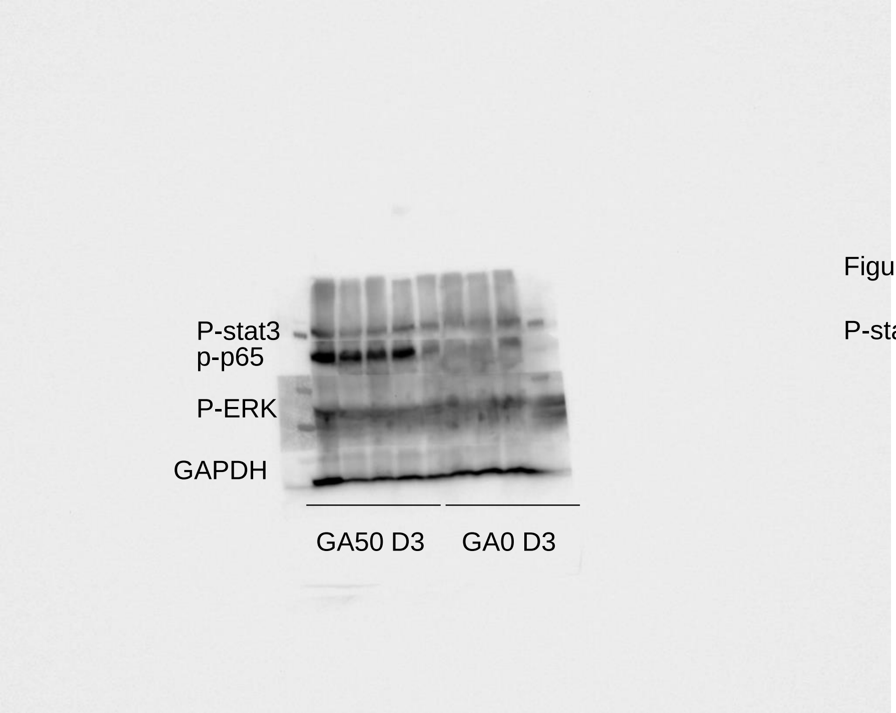

Figure 7E

P-stat3 and p-p65

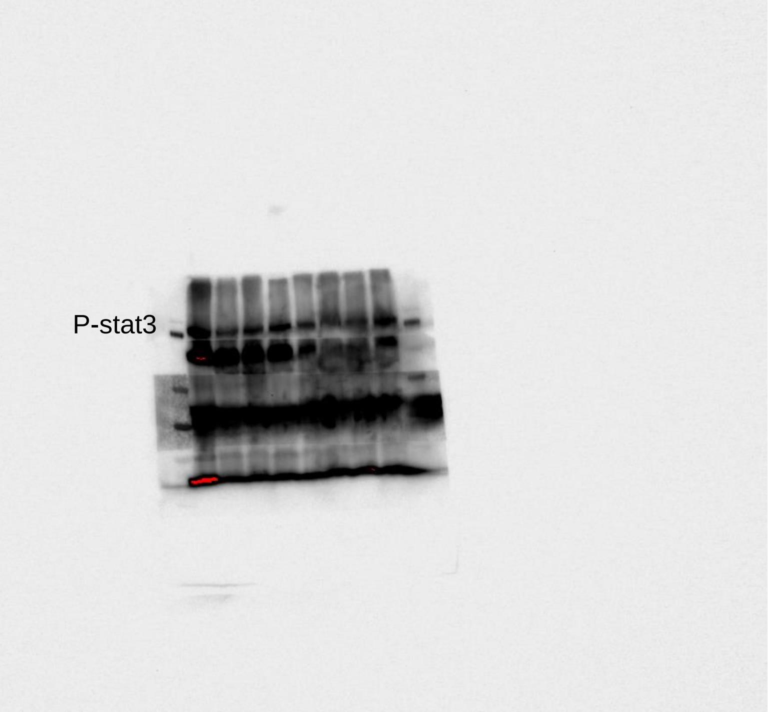

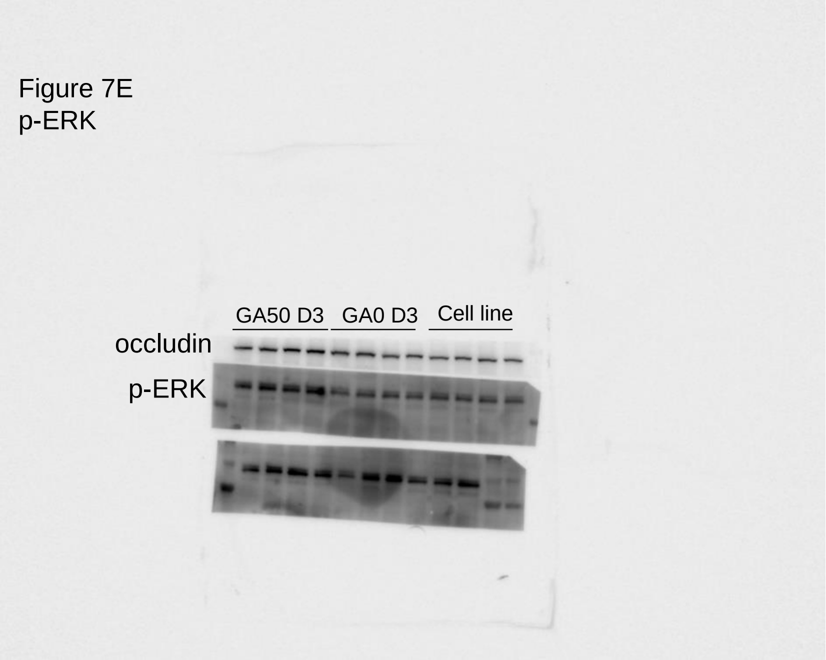

Supplement: Supplementary file 13 [file LSA-2022-01702_SdataF7.pdf]
