# Supplementary material for: Tannins amount determines whether tannase-containing bacteria are probiotic or pathogenic in IBD
Source: Life Sci Alliance. 2023 Feb 9;6(5):e202201702. doi: 10.26508/lsa.202201702 (PMC9911794; doi:10.26508/lsa.202201702)
Supplement: Supplementary file 14 [file LSA-2022-01702_TableS7.docx]

| **Gene name** | **Forward** | **Reverse** |
| --- | --- | --- |
| Human IL-6R | CCATTAGCCTGTCCGCCTC | TACTGGCACGGCTCGC |
| Human IL-1R1 | GGAGTCGCCAACTCAATTCG | CTTCAGAGGGTGCGTCTACC |
| Human CxCR2 | TCCTTCCTGGGTACAGTGCTAT | GCAGCTGTGACCTGCTGTTA |
| Human TNFRSF1B | GCACCGGGAGCTCAGATTCTT | ACCGAAAGGCACATTCCTCC |
| Human cxcl1 | CTGGCGGATCCAAGCAAATG | GCCCCTTTGTTCTAAGCCAG |
| Human cxcl2 | ATCAATGTGACGGCAGGGAA | CTCTGCTCTAACACAGAGGGAA |
| Mouse cxcl1 | TGAGCTGCGCTGTCAGTGCCT | AGAAGCCAGCGTTCACCAGA |
| Mouse cxcl2 | CAA GAACATCCAGAGCTT GAGTGT | GCC CTTGAG AGTGGCTAT GACTT |
| Mouse GAPDH | ACTCCACTCACGGCAAATTC | TCTCCATGGTGGTGAAGACA |
| Mouse ki67 | AGAAGTCCAGGTCTACAG | TCGTTGCTATTGCTAAGG |
| Mouse Occlaudin | GTGAGCTGTGATGTGTGTTGAGCT | GTGGGGAACGTGGCCGATATAATG |
| Mouse Zo-1 | GGGAGGGTCAAATGAAGACA | GGCATTCCTGCTGGTTACAT |
| Mouse Muc2 | GCTGACGAGTGGTTGGTGAATG | GATGAGGTGGCAGACAGGAGAC |
| Mouse Claudin-1 | TTCGCAAAGCACCGGGCAGATACA | GCCACTAATGTCGCCAGACCTGAAA |
| Human IL-6 | GCTGAAAAAGATGGATGCTT | GGCTTGTTCCTCACTACTCTC |
| Human IL1-beta | CCTGAGCTCGCCAGTGAAAT | TCGTGCACATAAGCCTCGTT |
| Human TNF-a | TCAGATCATCTTCTCGAACCCC | ATCTCTCAGCTCCACGCCAT |
| Mouse IL-6 | TCTATACCACTTCACAAGTCGGA | GAATTGCCATTGCACAACTCTTT |
| Mouse IL-1beta | CCTGAGCTCGCCAGTGAAAT | TCGTGCACATAAGCCTCGTT |
| Mouse TNF-a | TCCCAGGTTCTCTTCAAGGGA | GGTGAGGAGCACGTAGTCGG |
| Human Beta-actin | AGAGCTACGAGCTGCCTGAC | AGCACTGTGTTGGCGTACAG |
| Human CCNB1 | ACAGCTCTTGGGGACATTGG | GCACACAATTATTCTCAAGTTGTCT |
| Human PLK1 | TGACTCAACACGCCTCATCC | AGGAGACTCAGGCGGTATGT |
| Human CDC20 | GTTCGGGTAGCAGAACACCA | CCCCTTGATGCTGGGTGAAT |
| Human CCNB2 | TGCCTCCCCACTGATAGGAA | AAAGGGCACAATGAAGCACAC |
| Human CCNA2 | CAGAGGTTGGGAGTGGAAGAG | CTTCTTGGATGCCAGTCTTACTC |
| Human BuB1B | CTTCTGGGATGGGTCCTTCTG | GCTCTGAGGCAGCAATCTGT |
| Human PRKDC | AGCCATTGCCAGAGTACCAC | GGATCACTGGAGGTCATGGG |
| Human PTTG1 | CTCAGATGAATGCGGCTGTTAAGAC | TGCTCTTCAGGCAGGTCAAAA |
| Human BUB1 | ACTCACAGACACTGACGCTG | ATCTCCCTGGGTAGCTTCGT |
| Human CDC25C | TCAGAGGCCGTAACTTTGGC | CAGGCGAAGACTTGAGCAGA |
| Human TGFB2 | AGGTGCTCTGTGGGTACCTT | TGCAGCAGGGACAGTGTAAG |
| Lactobacillus murinus | AGAGTTAAGTGGCGAACGGG | TGTTTCCAAGTGTTATCCCCCT |
| Lactobacillus animalis | TGCGACCTTAACAAGTACCGA | ACCCGGTTGAAACCGATCTT |
| Bacteroides thetaiotaomicron | CGGTGTATCTGCCTGACGAT | ACTCCCGTTGGGAAAACCTG |
| Prevotella multisaccharivorax | TGCCCTTTAGGGTATGTGCAA | AGCAATTCAAGCCCGGGTAA |
| Universe QPCR-Eub341 | ACTCCTACGGGAGGCAGCAGT | ATTACCGCGGCTGCTGGC |

**Table S1-1 Primers for genes and bacteria**

**Table S1-2 Antibodies for WB**

| **Antibody** | **Catlog number** | **Company** |
| --- | --- | --- |
| Rabbit anti-E-Cadherin | A11509 | ABclonal |
| Mouse anti-GAPDH | AC033 | ABclonal |
| Rabbit anti-occludin | A12621 | ABclonal |
| Rabbit anti-Phospho-Stat3 S727 | AP0474 | ABclonal |
| Rabbit anti-Phospho-NF-kB p65/RelA-S536 | AP0475 | ABclonal |
| Rabbit anti-Phospho-ERK1-T202/Y204+ERK2-T185/Y187 | AP0974 | ABclonal |
| Rabbit anti-p53 | A0263 | ABclonal |
| Rabbit anti-phospho-p53-S46 | AP0476 | ABclonal |
